# ACADL plays a tumor-suppressor role by targeting Hippo/YAP signaling in hepatocellular carcinoma

**DOI:** 10.1038/s41698-020-0111-4

**Published:** 2020-03-25

**Authors:** Xiaofang Zhao, Wenhao Qin, Youhai Jiang, Zhishi Yang, Bo Yuan, Rongyang Dai, Hao Shen, Yao Chen, Jing Fu, Hongyang Wang

**Affiliations:** 1 Fudan University Shanghai Cancer Center, 200032 Shanghai, P. R. China; 2 International Cooperation Laboratory on Signal Transduction, Ministry of Education Key Laboratory on signaling Regulation and Targeting Therapy of Liver Cancer, Shanghai Key Laboratory of Hepato-biliary Tumor Biology, Eastern Hepatobiliary Surgery Hospital, Second Military Medical University, 200438 Shanghai, P. R. China; 3 Department of Biochemistry and Molecular Biology, Southwest Medical University, 646000 Luzhou, Sichuan P. R. China; 4 Cancer Research Center, The First Affiliated Hospital of USTC, Division of Life Sciences and Medicine, University of Science and Technology of China, Hefei, Anhui P. R. China; 5 Mengchao Hepatobiliary Hospital, Fujian Medical University, 350000 Fuzhou, Fujian P. R. China

**Keywords:** Cancer genetics, Cancer therapy

## Abstract

Long-chain acyl-CoA dehydrogenase (ACADL) is a mitochondrial enzyme that catalyzes the initial step of fatty acid oxidation, but the role of ACADL in tumor biology remains largely unknown. Here, we found that ACADL was frequently downregulated in hepatocellular carcinoma (HCC), and its low expression was significantly correlated with poor clinical prognosis of HCC patients. Restoring the expression of ACADL in HCC cells resulted cell cycle arrest and growth suppression through suppressing Hippo/YAP signaling evidenced by decreased YAP nuclear accumulation and downstream target genes expression. Reactivation of YAP by XMU-MP-1 diminished the inhibitory effect of ACADL on HCC growth. More importantly, the nuclear accumulation of YAP was negatively correlated with ACADL expression levels in HCC specimens, and YAP inhibitor verteporfin effectively suppressed growth of HCC organoids with low ACADL expression. Together, our findings highlight a novel function of ACADL in regulating HCC growth and targeting ACADL/Yap may be a potential strategy for HCC precise treatment.

## Introduction

Hepatocellular carcinoma (HCC), the major type of liver cancer, is the fifth most prevalent malignancy and the third leading cause of cancer-related death worldwide^1,2^. Rapid unlimited cell proliferation is the most malignant phenotype of cancer cells. As a highly heterogeneous tumor, although some regulators attributed to HCC progression has been identified, the molecular mechanisms underlying the rapid cell proliferation and growth of HCC cells are largely unknown^3^. There is an urgent need for more complete understanding of the molecular mechanisms involved in deregulated HCC cell proliferation, which could help improve therapeutic strategies.

Long-chain acyl-CoA dehydrogenase (ACADL) is a key enzyme in mitochondrial fatty acid oxidation, catalyzing the initial step for β-oxidation of long-chain fatty acyl-CoAs. It belongs to a family of four closely related, chain length-specific acyl-CoA dehydrogenases, which include very long-chain, long-chain, medium-chain, and short-chain acyl-CoA dehydrogenases (ACADVL, ACADL, ACADM, and ACADS, respectively)^4^. ACADL deficiency mice have severe hepatic and cardiac lipidosis, hypoglycemia, elevated serum-free fatty acids and hepatic insulin resistance caused by impaired fatty acid oxidation^5,6^.

Besides the regulation on fatty acid oxidation, the role of acyl-CoA dehydrogenase in tumor biology has been recently investigated. It was reported that ACADL contributed to the progression of prostate carcinoma and enhanced the malignant phenotypes of prostate cancer cells^7^. Victoria K. Hill and colleagues identified ACADL as one of the novel genes associated with tumorigenesis and poor survival in breast cancer by a genome-wide DNA methylation profiling of CpG islands^8^. A recent study revealed that loss of ACADL in HCC cells promoted tumor progression by regulating the PTEN pathway under hypoxia conditions^9^. Although studies have shown the correlation between ACADL and progression of some human cancers, the molecular mechanisms by which ACADL regulates HCC growth and progression are not well understood.

Herein, using gain-of function strategy, we showed that restoring ACADL expression in human HCC cells promoted cell cycle arrest, and inhibited cell proliferation and growth via decreasing YAP nuclear accumulation and target genes transcription. The negative correlation between YAP activation and ACADL expression was further detected in HCC specimens. In addition, suppressing YAP activity significantly inhibited growth of HCC organoids with low ACADL expression. Collectively, these studies suggest that ACADL functions as a tumor suppressor in HCC through regulating YAP activation.

## Results

### ACADL downregulation in HCCs predicts a poor prognosis

**Fig. 1:**
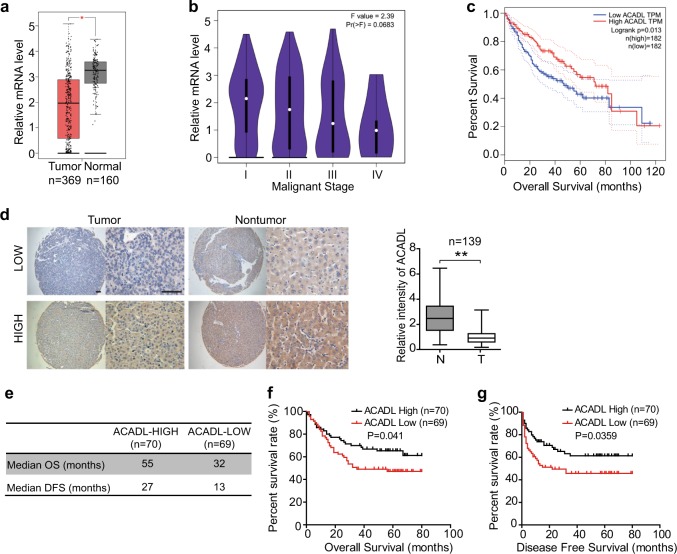
ACADL downregulation in HCCs predicts a poor prognosis. **a** Gene expression levels of ACADL in HCC (*n* = 369) and normal liver tissues (*n* = 160) from The Cancer Genome Atlas (TCGA) database. **b** Gene expression levels of ACADL in HCC patients from different stages in TCGA database. **c** Survival plots for groups with high and low expressions of ACADL expression in TCGA-LIHC cohort. **d** Expression of ACADL in HCC tissue microarray was tested by immunohistochemical staining. Representative micrographs showed low level and high level expression of ACADL in tumors tissues and adjacent nontumorous tissues. The measurement of all 139 specimens was quantified with the Image J software. Data are represented as mean ± S.D (**P < 0.01). Scale bar, 50 µm. **e** The median survival time of high (*n* = 70) and low (*n* = 69) ACADL expression groups. **f, g** Overall survival and Disease-free survival for the high and low ACADL expression groups were analyzed by Kaplan-Meier survival analysis.

To determine the expression status of ACADL in HCC, we queried The Cancer Genome Atlas (TCGA) hepatocellular carcinoma database for the expression variations between tumor tissues and normal liver tissues. Our analysis revealed that the mRNA levels of ACADL dramatically decreased in HCC tissues (*n* = 369) compared with normal livers (*n* = 160) (Fig. 1a). Notably, the tumor stage plot analysis from TCGA database showed ACADL expression was gradually decreased as HCC progressed to a higher clinical stage (Fig. 1b). Moreover, HCC patients with low ACADL expression had poorer overall survival than those with high ACADL expression (Fig. 1c).

To further investigate the expression and clinical significance of ACADL in HCC, we checked the mRNA and protein levels of ACADL in clinically and pathologically characterized HCC tissues and case-matched normal tissues from our hospital. The real-time PCR and western blot results showed that ACADL substantially downregulated in tumors compared with the adjacent nontumorous tissues in both mRNA and protein levels (Supplementary Fig. 1). We also examined ACADL expression in another independent 139 HCC samples on a tissue microarray (TMA) by immunohistochemical staining. Consistently, the average expression level of ACADL protein was significantly lower in HCC tissues than in adjacent nontumorous tissues (Fig. 1d). According to the immunohistochemical results of ACADL staining in tumor sections, the total 139 HCC samples were divided into low ACADL expression group (*n* = 69) and high expression group (*n* = 70). Intriguingly, the low ACADL expression group had increased serum AFP levels (*P* = 0.01), larger tumor size (*P* = 0.036), promoted vascular invasion (*P* = 0.004), and recurrence (*P* = 0.002) (Table 1). Kaplan-Meier survival analyses indicated that patients with low ACADL expression had much shorter overall survival times (OS, Fig. 1e, f) and disease-free survival times (DFS, Fig. 1e, g). These together suggested that ACADL expression is a valuable predicting factor for malignant progression of HCC.

**Table 1:** Relationship between intratumoral ACADL expression and clinicopathologic features.

		ACADL density		
Variable	All cases	Low (*n* = 69)	High (*n* = 70)	*P*-value
Age (years)				0.018
≤50	72	43	29	
>50	67	26	41	
Gender				0.426
Female	16	6	10	
Male	123	63	60	
HBsAg				0.277
Positive	113	59	54	
Negative	26	10	16	
AFP (ng⁄ mL)				0.010
<20	35	11	24	
≥20	104	58	46	
Cirrhosis				0.235
Yes	76	34	42	
No	63	35	28	
Tumor size (cm)				0.036
<6	64	26	38	
≥6	75	43	32	
TNM Stage				0.546
I–II	107	55	52	
III–IV	32	14	18	
Edmondson				0.275
I–II	8	2	6	
III–IV	131	67	64	
Vascular invasion				0.004
Yes	73	45	28	
No	66	24	42	
Involucrum				1.000
Complete	54	27	27	
Incomplete or absent	85	42	43	
Recurrence				0.002
Yes	71	44	27	
No	68	25	43	

Patients with HCC were divided into ACADL Low expression group (whose final density was lower than the median) and High expression group (whose final density was higher than the median). The patient and disease profiles in each group were compared.

### Restored ACADL suppresses HCC cell growth in vitro and in vivo

**Fig. 2:**
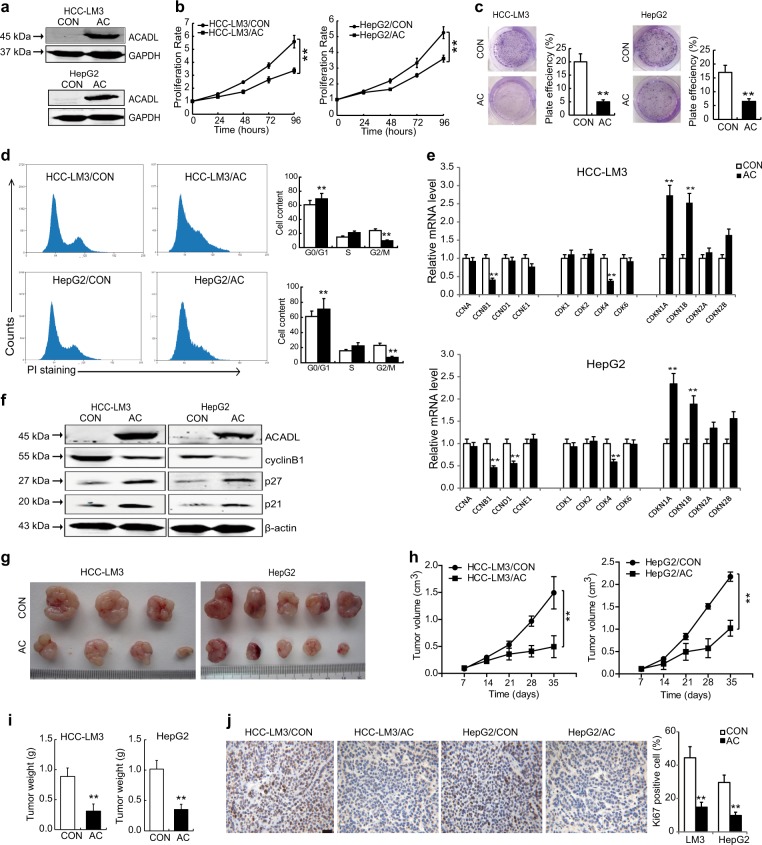
Restoring expression of ACADL suppresses HCC cell growth in vitro and in vivo. **a** Protein levels of ACADL in the HCC-LM3 and HepG2 cells infected with lentivirus encoding ACADL and respective controls were detected by western blot. **b** Cell proliferation was detected by CCK-8 assay at various time points (*P < 0.05). **c** Representative image and quantification of colony formation in ACADL overexpression and control cells (**P < 0.01). **d** Cell cycle of HCC-LM3/AC, HepG2/AC and the respective control cells were analyzed by flow cytometry (**P < 0.01). **e** The mRNA levels of cell cycle related genes were determined by real-time PCR (**P < 0.01). **f** The expression level of cyclinB1, p27, p21, ACADL were compared by western blotting, and β-actin was used as a loading control. **g** The HCC-LM3/AC, HepG2/AC, and control cells were injected subcutaneously into nude mice. Representative image of the xenograft tumors obtained from the indicated groups at the endpoint of the experiments (day 35). **h** Tumor volumes were measured and presented as mean ± SD. (**P < 0.01). **i** The final tumor weight of each group (**P < 0.01). **j** The expression of Ki67 in the tumor sections from each group were detected by immunohistochemical staining. Scale bar, 50 µm.

In order to evaluate the effect of ACADL on HCC cells, we employed lentivirus encoding ACADL to establish ACADL stable cell lines with HCC-LM3 and HepG2 cells (named as HCC-LM3/AC and HepG2/AC), with GFP as control (named as HCC-LM3/CON and HepG2/CON). ACADL expression in the infected cells was confirmed by western blot (Fig. 2a). Re-expression of ACADL substantially inhibited proliferation (Fig. 2b) and colony formation (Fig. 2c) of HCC cells. Flow cytometry analysis further showed that restored ACADL in HCC cells induced G1 phase arrest and decreased percentage of G2/M population (Fig. 2d). Consistently, the key regulatory factors of cell cycle progression^10^, including cyclinB1, cyclinD1, and CDK4 were significantly decreased, while the negative regulators of cell cycle progression, including CDKN1A (P21) and CDKN1B (P27) increased in HCC-LM3/AC and HepG2/AC cells compared with the controls (Fig. 2e, f). These results demonstrated that ACADL re-expression inhibited proliferation of HCC cells. However, restored ACADL had no significant effect on cell invasion or stem-like properties of HCC cells (Supplementary Fig. 2).

To assess the role of ACADL in tumor growth in vivo, HCC-LM3/AC, HepG2/AC, and control cells were injected subcutaneously into nude mice. The data revealed that tumor growth were remarkably suppressed in ACADL re-expressed groups compared with control groups (Fig. 2g, h), and the ACADL re-expressed groups had much lower tumor weight than the control groups at the end of observation (Fig. 2i). The immunohistochemical staining showed an evident decrease of Ki67 expression in the ACADL re-expressed tumors (Fig. 2j). Furthermore, a negative correlation between mRNA levels of ACADL and Ki67 (a marker for proliferation), or cyclinB1, CDK4 were also observed in HCC tissues from TCGA. In contrast, ACADL showed positive correlation with cell cycle inhibitors, including CDKN1A, CDKN1B and CDKN2C, indicating suppressed cell proliferation by ACADL expression in vivo (Supplementary Fig. 3).

Considering that ACADL is a key enzyme of fatty acid oxidation, ACADL-mediated repression of HCC cell proliferation and growth could be due to the abnormal fatty acid metabolism. We therefor used etomoxir, a fatty acid β-oxidation inhibitor to treat HCC-LM3/AC, HepG2/AC cells and control cells. Although etomoxir treatment significantly inhibited cell proliferation of both control and ACADL re-expressing cells, HCC-LM3/AC and HepG2/AC cells still showed inhibited proliferation and colony formation compared with the control cells (Supplementary Fig. 4). These data together suggested that restored ACADL suppressed HCC cell growth in a fatty acid oxidation independent manner.

### ACADL inhibits the activation of Hippo/YAP pathway in HCC cells

**Fig. 3:**
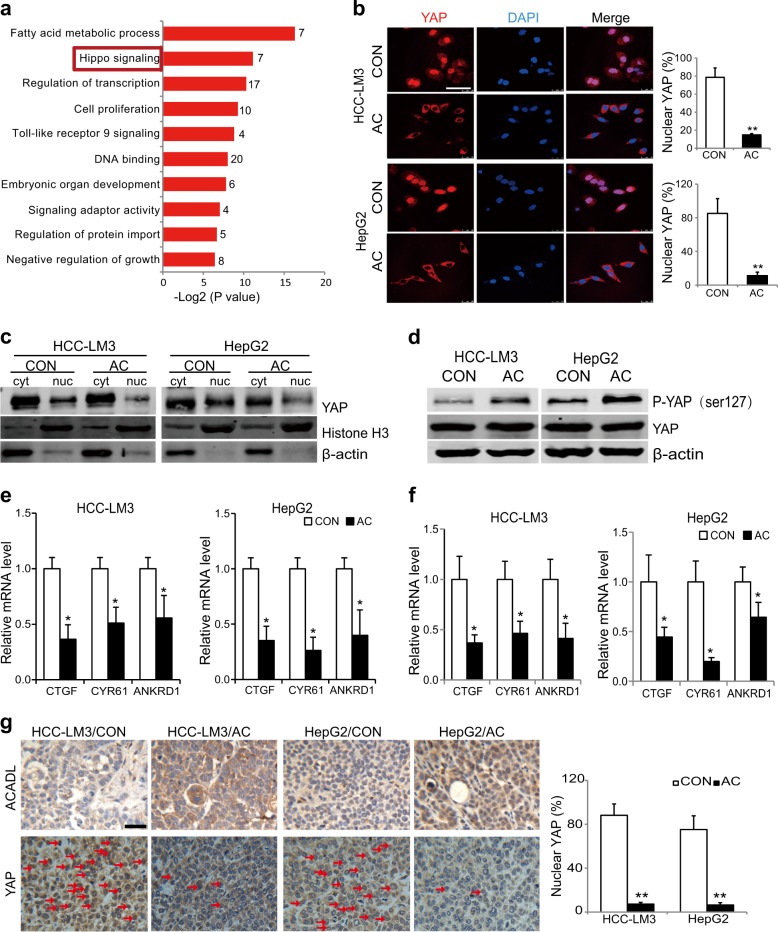
Restored expression of ACADL suppressed the Hippo/YAP signaling pathway in HCC cells. **a** Differentially expressed genes between HCC-LM3/AC and HepG2/AC cells and their control cells were used to perform pathway analysis based on the KEGG database. Differentially expressed gene numbers of each pathway were marked next to the bars. **b** Representative images and quantification of immunofluorescent staining for YAP. (**P < 0.01) **c** Cytosolic and nuclear proteins from indicated cells were separated to detect expression of YAP by western blotting. Histone H3 and β-actin were used as a loading control. **d** Cell lysates from indicated cells were subjected to immunoblot for phosphorylated YAP (Ser127) and total YAP. **e** The mRNA levels of CTGF, CYR61, and ANKRD1 in ACADL overexpression and control cells. (*P < 0.05) **f** The mRNA levels of CTGF, CYR61 and ANKRD1 in xenograft tumors of indicated groups. (*P < 0.05). **g** Representative images of immunohistochemical staining for ACADL and YAP in tumor sections from xenograft tumors. YAP nuclear localization ratios were quantified (**P < 0.01). Scale bar, 50 µm.

To explore the mechanism underlying ACADL-mediated inhibition of cell growth, we performed RNA sequence analysis of HCC-LM3/AC, HepG2/AC cells and their control cells. According to the following criteria: Fold Change >1.2 or <0.83 and a false discovery rate (FDR) < 0.2, 107 differentially expressed genes (DEGs) were identified. Pathway analysis revealed that besides fatty acid metabolic process, the DEGs were also significantly enriched in Hippo pathway, implying the involvement of Hippo signaling in ACADL-mediated HCC growth inhibition (Fig. 3a). Considering that transcription cofactor Yes-associated protein (YAP) is a major downstream effector of the Hippo pathway, the YAP activity were analyzed in ACADL restored and control cells. The immunofluorescence results exhibited decreased nuclear localization of YAP in ACADL restored cells (Fig. 3b). In addition, cytoplasmic/nuclear protein extraction assay also indicated less nuclear accumulation of YAP in ACADL re-expressed cells than control cells (Fig. 3c). It is known that phosphorylation of YAP on the residue serine127 by MST1/2 and LATS1/2 sequesters YAP in the cytosol and limits transcriptional activity^11^. As expect, ACADL re-expression promoted the phosphorylation of YAP in HCC cells (Fig. 3d). Furthermore, decreased mRNA levels of canonical YAP target genes were detected in ACADL restored HCC cells, including *Connective Tissue Growth Factor* (*CTGF*), *Cysteine Rich Angiogenic Inducer 61* (*CYR61*), and *Ankyrin Repeat Domain 1* (*ANKRD1*) (Fig. 3e). Consistently, re-expression of ACADL resulted in decreased YAP nuclear accumulation and YAP target genes expression in HCC xenograft tumors (Fig. 3f, g), that further confirmed the inhibitory effect of ACADL on YAP activity.

### YAP reactivation diminishes ACADL-mediated growth inhibition

**Fig. 4:**
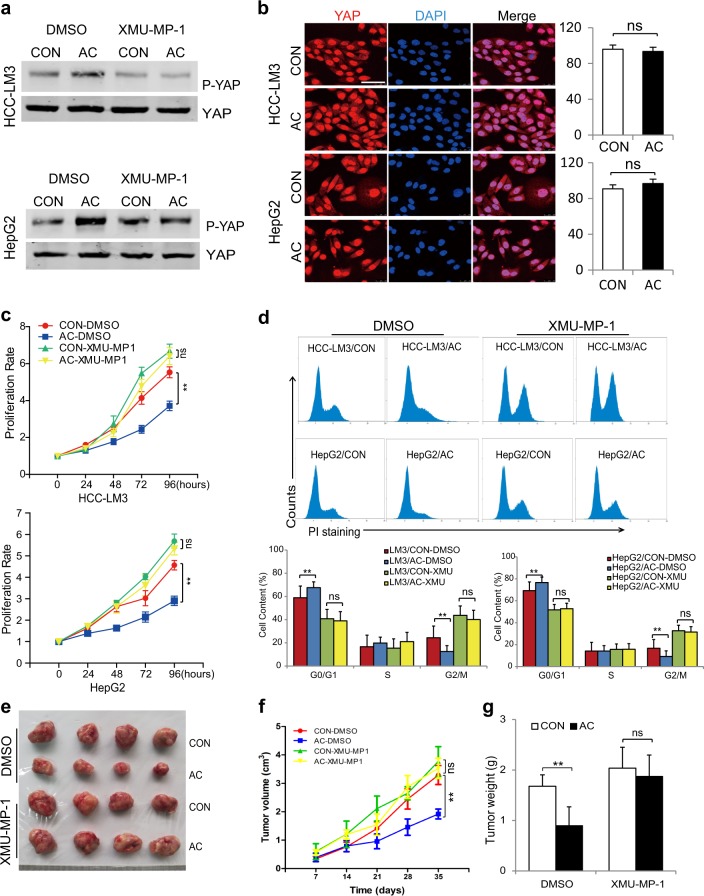
Reactivating YAP diminished ACADL-mediated HCC cell growth inhibition. **a** Cell lysates from indicated cells treated with XMU-MP-1 (1 µM) or DMSO were subjected to immunoblot for phosphorylated YAP (Ser127) and total YAP. **b** Representative images and quantification of immunofluorescent staining for YAP distribution after cells were treated with XMU-MP-1 (1 µM) for 12 h (ns no significance). Scale bar, 50 µm. **c** The HCC-LM3/AC, HepG2/AC, and control cells were treated with XMU-MP-1 (1 µM) or DMSO for indicated time. Cell proliferation was detected by CCK-8 assay at various time points (**P < 0.01, ns no significance). **d** Cell cycle was analyzed by flow cytometry after indicated cells treated with XMU-MP-1 (1 µM) or DMSO for 12 h. (**P < 0.01, ns no significance). **e** The HCC-LM3/AC and control cells were injected subcutaneously into nude mice. A week later, the mice were administrated with XMU-MP-1 (1 mg/kg) or DMSO for indicated time. Representative image of the xenograft tumors obtained from the indicated groups. **f** Tumor volumes were measured and presented as mean ± SD (**P < 0.01, ns no significance). **g** The final tumor weight of each group (**P < 0.01, ns = no significance).

Next, to further address if YAP is involved in ACADL-regulated cancer cell proliferation, we applied XMU-MP-1, an activator of Hippo pathway, which can promote YAP nuclear translocation and reactivate YAP^12^. XMU-MP-1 administration reversed ACADL-induced YAP phosphorylation and consequently cytosol accumulation (Fig. 4a, b). In addition, XMU-MP-1 treatment also eliminated ACADL-mediated cell proliferation suppression and cell cycle arrest in HCC-LM3 and HepG2 cells (Fig. 4c, d). More importantly, ACADL-suppressed tumor growth in nude mice was diminished after XMU-MP-1 exposure (Fig. 4e–g). Taken together, these results imply that YAP repression is required for ACADL-induced inhibition of HCC cell growth.

### ACADL expression are correlated with YAP activation in human HCC tissues

**Fig. 5:**
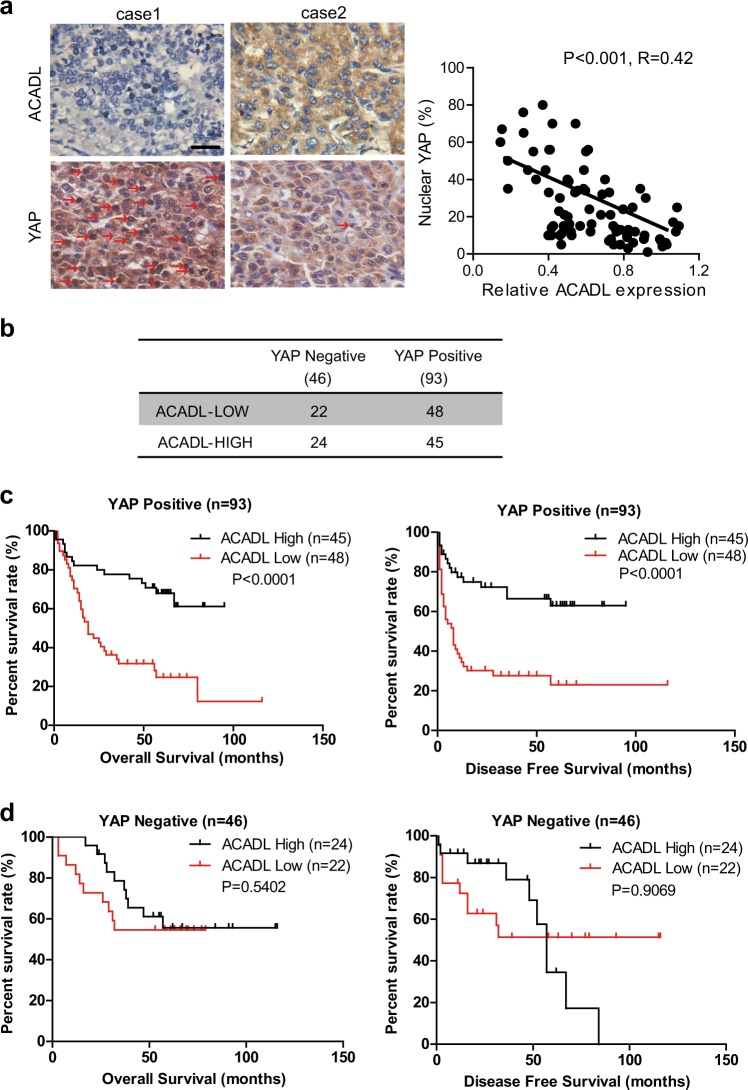
ACADL expressions are correlated with YAP activation in human HCC tissues. **a** Representative images of immunohistochemical staining for ACADL and YAP in HCC specimens. The expression intensity of ACADL and nuclear localization of YAP in 93 HCC specimens were quantified and the correlation was calculated. Scale bar, 50 µm. **b** The 139 HCC specimens were divided into different groups according to the expression levels of YAP and ACADL. **c** The overall survival and disease-free survival rates were compared between the ACADL-low expression and ACADL-high expression groups in the YAP-positive group. **d** The overall survival and disease-free survival rates were compared between the ACADL-low expression and ACADL-high expression groups in the YAP negative group.

To extend our in vitro results to humans, the expression of ACADL and YAP activation in 139 HCC samples on TMA were examined by immunohistochemistry. We found that 93 HCC samples had different levels of total YAP expression (YAP-Positive) and 46 HCC samples showed very low or none detectable YAP expression (YAP Negative). Quantification analysis of the 93 HCC specimens (YAP-Positive) revealed a strong negative correlation between ACADL expression density and YAP nuclear localization, but no correlation with total YAP expression (Fig. 5a, Supplementary Fig. 5). According to the ACADL expression level, the 93 HCCs with YAP expression were divided into ACADL-low-density group (*n* = 48) and high-density group (*n* = 45) (Fig. 5b). There was a significant difference in both overall survival (*P* < 0.0001) and disease-free survival (*P* < 0.0001) between ACADL-low and high groups (Fig. 5c). However, ACADL-low-density group (*n* = 22) and high-density group (*n* = 24) in HCCs with no YAP expression (YAP Negative) had no remarkable difference in patient survival (Fig. 5d). These data reflected that only in patients whose tumors had YAP expression, low ACADL expression is a powerful predictor of poor prognosis, which further supported a critical role of YAP in ACADL-mediated HCC suppression.

### YAP-targeted drug represses growth of HCC organoids with low expression of ACADL

**Fig. 6:**
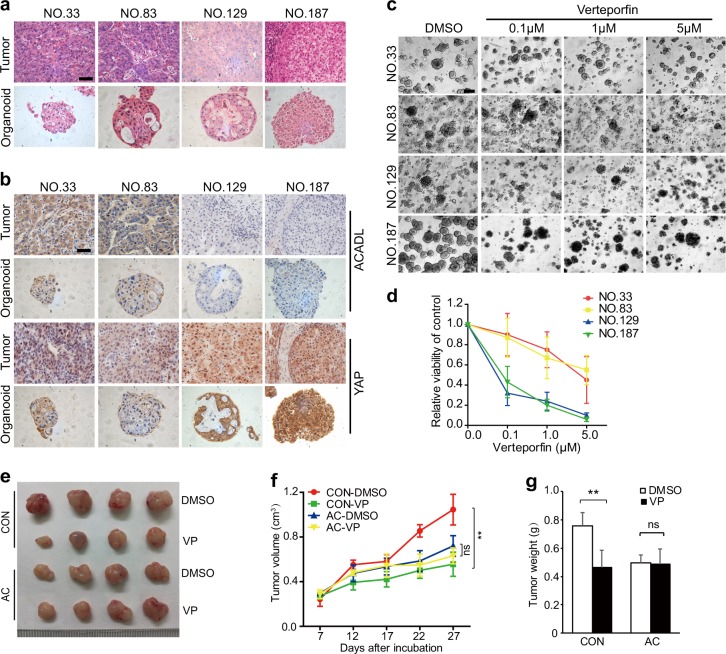
YAP-targeted drug represses growth of HCC organoids with low expression of ACADL. **a** H&E staining of the primary HCC tissue specimens (NO.33, NO.83, NO.129, and NO.187) and organoids derived from them. Scale bars, 50 µm. **b** Immunohistochemistry for ACADL and YAP in the primary tissue of HCC and organoids. Scale bars, 50 µm. **c** Bright-field images of HCC organoids after treated with DMSO or verteporfin of indicated concentration. Scale bars, 200 µm. **d** Dose-response curves after 4 days of treatment of organoids with verteporfin. **e** The HCC-LM3/AC and control cells were injected subcutaneously into nude mice. A week later, the mice were administrated with verteporfin (VP, 10 mg/kg) or DMSO for indicated time. Representative image of the xenograft tumors obtained from the indicated groups. **f** Tumor volumes were measured and presented as mean ± SD (**P < 0.01, ns no significance). **g** The final tumor weight of each group (**P < 0.01, ns no significance).

To further verify the importance of ACADL-YAP signaling to the growth of cancer cells in HCC patients, tumor tissue derived organoids were established. Firstly, H&E staining showed that HCC organoids well recapitulated the histopathological features of the original tissues (Fig. 6a). Then the expression of ACADL and YAP activation were examined in HCC organoids and original tumor tissues. The HCC organoids displayed consistent expression patterns of ACADL and YAP with original tissues (Fig. 6b). We next investigated the correlation between ACADL expression and therapeutic effect of YAP-targeted drugs in HCC organoids. HCC organoids with high ACADL (NO.33 and NO.83) and low ACADL (NO.129 and NO.187) expression were treated with YAP inhibitor verteporfin, that pharmacologically disrupts TEAD-YAP interactions^13^. As expected, organoids with low expression of ACADL were sensitive to verteporfin, whereas the organoids with high expression levels of ACADL were insensitive to verteporfin (Fig. 6c, d). These findings indicate that verteporfin could be a potential therapeutic drug for HCC patients harboring low expression levels of ACADL. The pharmacological role of verteporfin in vivo was investigated in HCC-LM3 xenograft model. The results showed that verteporfin markedly reduced tumor growth in control groups, whereas the tumors with re-expressed ACADL had not responsive to verteporfin treatment, which support the data in HCC organoids (Fig. 6e–g).

## Discussion

ACADL, a member of the acyl-CoA dehydrogenases superfamily, is responsible for catalyzing the initial step for the β-oxidation of long-chain fatty acyl-CoAs and has recently been reported to play roles in tumor progression^5^. Jian-Min Wu and his colleagues found that the expression of ACADL was drastically downregulated in the HCV-associated HCC^14^. A recent paper demonstrated that the expression of ACADL was downregulated by HIF-1α under hypoxic conditions in human HCC cells, and decreased ACADL expression led to cancer progression through promoting accumulation of unsaturated fat^9^. Here, we analyzed a retrospective cohort of 139 HCC specimens from our hospital. Consistent with their findings, our data revealed a distinct decreased expression of ACADL in HBV-associated HCC and the low ACADL expression correlated with more aggressive clinical characteristics and poor prognosis. Additionally, studies from the TCGA database suggested ACADL expression is positively correlated with clinical HCC stage and patient survival. These data strongly indicated that ACADL could serve as a novel prognostic marker for HCCs with different risk factors. However, our studies using gain of function strategies uncovered that ACADL possessed tumor-suppressive effects on HCC cells under normal conditions. More importantly, ACADL-mediated suppressive effect on HCC cell growth was not diminished by etomoxir, the fatty acid oxidation inhibitor, suggesting the existence of other mechanisms independent of its function in fatty acid oxidation.

Hippo/YAP signaling is a conserved regulator that controls cell growth, apoptosis, organ size and tumorigenesis^15^. The Hippo pathway comprises several tumor suppressors, including Mst1/2, Sav1/WW45, Lats1/2, and Mob1, which act as kinases that phosphorylate and inactivate of YAP. YAP acts as the transcriptional co-activator to promote the expression of their target genes involved in proliferation and survival^11,16^. Accumulating studies have implicated that Hippo/YAP signaling pathway played a vital role in the tumorigenesis of HCC^17–19^. It has been reported that overexpression of YAP in mice liver leads to expanded liver size and the Mst1/2 deficiency in the liver results in activation of YAP, and liver cancer^18,20^. In this study, using RNA sequence analysis, we found that several downstream genes in Hippo pathway were significantly downregulated in ACADL-overexpressing HCC cells, and these cells exhibited restricted nuclear localization and transcriptional activity of YAP. Moreover, reactivating YAP by Mst1/2 inhibitor XMU-MP-1 counteracted the inhibitory effect of ACADL on cell growth, cell cycle progression and tumorigenesis of HCC cells.

Here, we also found a negative association between YAP nuclear localization and the expression intensity of ACADL in HCC samples, which were consistent with our in vitro data. More importantly, ACADL acted as a more powerful prognostic biomarker in HCC patients with YAP expression than those with no YAP expression, indicating that low ACADL expression promoted HCC growth and progression mainly through activating YAP. Huang et al. demonstrated that depletion of ACADL promotes HCC progression by reduction of PTEN. Since there is a crosstalk between YAP and PTEN, that YAP suppresses PTEN via regulating miR-29^21^, it is possible that ACADL may suppress HCC cell proliferation via the YAP-PTEN signaling. We currently do not know how ACADL regulates YAP activation. It was reported that downregulation of ACADL reduces ROS and increases cellular fatty acids, and ACADL itself directly produces ROS^9,22^. In addition, it has been reported that ROS or fatty acids overload inhibited YAP activation through regulating LATS1/2^23,24^. Thus, it is possible that ACADL may regulate the YAP activity through modulating ROS or fatty acid levels. Further investigations on these topics may help us gain a full understanding of mechanisms behind ACADL, YAP signaling and the HCC progression.

Given the important role of Hippo/YAP signaling in HCC, numerous researches attempted to explore YAP targeting drugs. Many researchers have discovered numerous small molecules that can regulate Hippo/YAP pathway components; however, successful YAP-targeted anti-tumor drugs are rare. Verteporfin, firstly designed for use in phototherapy, binds to YAP to inhibit the interaction of YAP with TEAD and its transcriptional activity^13,25^. Verteporfin has been reported to inhibit cell proliferation or induce apoptosis in several tumors, including leukemia, pancreatic cancer, and lung cancer^26–28^. Here, we found that verteporfin effectively inhibited the growth of HCC organoids with low expression of ACADL, while had faint effect on organoids with high ACADL expression, indicating that verteporfin could be used as a precise medicine in human HCC therapy.

In summary, our findings identify the tumor-suppressive effects of ACADL through inhibiting the Hippo/YAP signaling, and provide proof of principle that inhibiting YAP activation will be a viable strategy for HCC patients with low ACADL expression.

## Methods

### Clinical samples

All paired samples of primary HCC, their corresponding nontumorous liver tissues and samples in tissue microarray (TMA) were obtained during surgical resection at Department of Liver Surgery, Eastern Hepatobiliary Surgery Hospital, Second Military Medical University (SMMU), Shanghai, China. 139 HCC samples of TMA were collected from 2003 to 2011 and follow-up ended in May 2011. Patient age ranged from 22 to 78 years, with a median age of 50 years. Written informed consent was obtained from all participants prior to the start of the study. All studies were approved by the Ethical Committee of the SMMU and performed in accordance with relevant regulations and guidelines.

### Plasmid constructs and stable cell line establishment

Full length human ACADL or green fluorescent protein (GFP) CDS sequence was inserted into pLenti-CMV-3FLAG lentiviral vector (OBiO Technology, Shanghai). The lentiviral vectors were transfected into the HCC cells with a multiplicity of infection (MOI) 20. After 12 h the original medium was replaced with fresh medium.

### Immunohistochemical staining

Tissue samples embedded in 4% paraffin were cut into 5 μm sections and stained following the routine protocol. The primary antibodies were the following: anti-ACADL (1:200, HPA011990, Sigma-Aldrich, Gillingham, Dorset, UK), anti-Ki67 (1:100, ab15580, Abcam, Cambridge, UK), and anti-YAP (1:200, 14074, Cell Signaling Technology, Boston, MA, USA). The staining intensity was quantifed with Image J software.

### Quantitative real-time PCR

Total RNA was extracted from tissues and HCC cell lines using Trizol reagent (Invitrogen, Carlsbad, CA, USA) according to the manufacturer’s instructions. RNA was reversely transcribed into cDNA using Superscript III RT (Invitrogen, Carlsbad, CA, USA) and random primers. Quantitative RT-PCR was subsequently performed with SYBR Premix Ex Taq (Takara, Otsu, Shiga, Japan) using an ABI PRISM 7300HT Sequence Detection System (Applied Biosystems, Foster City, CA). Primer sequences were listed in supplementary materials.

### Western blot analysis

Human Tissue Specimen or whole mouse liver tissue were homogenized in Triton lysis buffer (20 mM Tris, pH 7.4, 137 mM NaCl, 10% glycerol, 1% Triton X-100, 2 mM EDTA, 1 mM PMSF, 10 mM NaF, 5 mg/ml aprotinin, 20 mM leupeptin, and 1 mM sodium orthovanadate) and centrifuged at 13,000 × *g* for 15 min. Protein extracts were subjected to SDS-PAGE. Primary antibodies were following: anti-ACADL (1:1000, HPA011990, Sigma-Aldrich), anti-cyclin B1 (1:1000, 4135, Cell Signaling Technology), anti-p27 (1:1000, 3698, Cell Signaling Technology), anti-p21 (1:1000, 2947, Cell Signaling Technology), anti-YAP (1:1000, 14074, Cell Signaling Technology), anti-Phospho-YAP (Ser127) (1:1000, 13008, Cell Signaling Technology), anti-Histone H3 (1:1000, 1791, Abcam) and anti-β-actin (1:1000, 60008, Proteintech Group).

### Clone formation assay and cell proliferation assay

For colony formation assay, cells were plated in 6-well plates in a density of 4000/well and XMU-MP-1 (1 mM) or DMSO was added. After 14 days, the number of colonies was counted and representative wells were photographed. Cell proliferation assay was performed with the Cell Counting Kit 8 assay (Dojindo, Kumamoto, Japan) according to manufacturer’s protocols.

### Cell cycle analysis by flow cytometry

Cells were fixed overnight, suspended in phosphate-buffered saline, and stained with propidium iodide (PI) in the dark for 30 min. The DNA content was measured by fluorescence-activated cell sorting (FACS) on a Becton-Dickinson FAC Scan flow cytometry system.

### Fluorescence microscopy

Cells were plated onto culture slides, fixed with 4% paraformaldehyde, and permeabilized with 0.1% Triton X-100. After blocking, cells were incubated with primary YAP antibodies and then Alexa Fluor 555 anti-rabbit IgG secondary antibody (Invitrogen). Nuclei were visualized by staining with DAPI.

### Cytoplasmic and nuclear protein extraction

The cytoplasmic and nuclear proteins of HCC-LM3 and HepG2 cells were extracted using Nuclear and Cytoplasmic Extraction Reagents (Thermo Fisher, UK) according to the manufacturer’s protocol.

### HCC organoids culture

HCC tumor tissue derived organoids were established as follows. Briefly, HCC tissues were minced and incubated at 37 °C with Collagenase IV (Roche, Burgess Hill, UK) digestion solution for 30 min to 1 h. Digestion was stopped by adding DMEM with 10% FES, and the suspension was then filtered through a 100 µm nylon cell strainer and spun for 5 min at 300 × *g*. The pellet was washed in cold Advanced DMEM/F12 (Invitrogen), and then mixed with matrigel (BD Transduction Laboratories, NJ, USA). Cells were seeded in 6-multiwell suspension plate. After matrigel had solidified, the samples were cultured in the medium described by Laura Broutier^29^.

### Tumorigenesis in nude mice

Animal xenograft assays were conducted with 6–8-week-old male nude mice. 5 × 10^6^ indicated cells were injected subcutaneously in the right flank of each mouse. Seven days after inoculation, mice were randomly divided into different groups (*n* = 6). XMU-MP-1 (Selleck Chemicals, Houston, TX, USA) was administered twice a week at a dose of 1 mg/kg by intraperitoneal injection for 3 weeks. Verteporfin (Selleck Chemicals, Houston, TX, USA) was administered every second day at a dose of 10 mg/kg by intraperitoneal injection for 2 weeks. The control group mice were administrated with DMSO for indicated time. Tumor size was measured weekly using a caliper, and tumor volume was calculated by the formula: (width)^2^ × length/2. After 4–6 weeks, mice were sacrificed and tumors were excised. All animal experiments were approved by the Ethical Committee of the Second Military Medical University (SMMU) and conducted according to the SMMU Animal Care Facility guidelines.

### Statistical analysis

Data analysis was carried out by the SPSS software (version 16; SPSS). Each experiment was in triplicate at least and values were presented as mean ± SD. Statistic differences were calculated using Chi-square test and Student’s *t*-test. Values of *P* < 0.05 were considered statistically significant.

### Reporting summary

Further information on experimental design is available in the Nature Research Reporting Summary linked to this article.

## Supplementary information

Supplementary material

Reporting Summary

## Data Availability

The authors declare that the data supporting our findings are included in the paper and its supplemental information files.

## References

[1] Torre LA (2015). Global cancer statistics, 2012. CA Cancer J. Clin..

[2] Llovet JM (2016). Hepatocellular carcinoma. Nat. Rev. Dis. Prim..

[3] Llovet JM, Montal R, Sia D, Finn RS (2018). Molecular therapies and precision medicine for hepatocellular carcinoma. Nat. Rev. Clin. Oncol..

[4] Kurtz DM, Tolwani RJ, Wood PA (1998). Structural characterization of the mouse long-chain acyl-CoA dehydrogenase gene and 5’ regulatory region. Mamm. Genome.

[5] Kurtz DM (1998). Targeted disruption of mouse long-chain acyl-CoA dehydrogenase gene reveals crucial roles for fatty acid oxidation. Proc. Natl Acad. Sci. USA.

[6] Zhang D (2007). Mitochondrial dysfunction due to long-chain Acyl-CoA dehydrogenase deficiency causes hepatic steatosis and hepatic insulin resistance. Proc. Natl Acad. Sci. USA.

[7] Xie BX (2011). Analysis of differentially expressed genes in LNCaP prostate cancer progression model. J. Androl..

[8] Hill VK (2011). Genome-wide DNA methylation profiling of CpG islands in breast cancer identifies novel genes associated with tumorigenicity. Cancer Res..

[9] Huang J (2014). HIF-1-mediated suppression of acyl-CoA dehydrogenases and fatty acid oxidation is critical for cancer progression. Cell Rep..

[10] Li J, Poi MJ, Tsai MD (2011). Regulatory mechanisms of tumor suppressor P16(INK4A) and their relevance to cancer. Biochemistry.

[11] Zhao B (2007). Inactivation of YAP oncoprotein by the Hippo pathway is involved in cell contact inhibition and tissue growth control. Genes Dev..

[12] Fan F (2016). Pharmacological targeting of kinases MST1 and MST2 augments tissue repair and regeneration. Sci. Transl. Med..

[13] Liu-Chittenden Y (2012). Genetic and pharmacological disruption of the TEAD-YAP complex suppresses the oncogenic activity of YAP. Genes Dev..

[14] Wu JM, Skill NJ, Maluccio MA (2010). Evidence of aberrant lipid metabolism in hepatitis C and hepatocellular carcinoma. HPB (Oxf.).

[15] Pan D (2010). The hippo signaling pathway in development and cancer. Dev. Cell.

[16] Huang J, Wu S, Barrera J, Matthews K, Pan D (2005). The Hippo signaling pathway coordinately regulates cell proliferation and apoptosis by inactivating Yorkie, the Drosophila Homolog of YAP. Cell.

[17] Harvey KF, Zhang X, Thomas DM (2013). The Hippo pathway and human cancer. Nat. Rev. Cancer.

[18] Zhou D (2009). Mst1 and Mst2 maintain hepatocyte quiescence and suppress hepatocellular carcinoma development through inactivation of the Yap1 oncogene. Cancer Cell.

[19] Yimlamai D, Fowl BH, Camargo FD (2015). Emerging evidence on the role of the Hippo/YAP pathway in liver physiology and cancer. J. Hepatol..

[20] Camargo FD (2007). YAP1 increases organ size and expands undifferentiated progenitor cells. Curr. Biol..

[21] Tumaneng K (2012). YAP mediates crosstalk between the Hippo and PI(3)K-TOR pathways by suppressing PTEN via miR-29. Nat. Cell Biol..

[22] Zhang Y, Bharathi SS, Beck ME, Goetzman ES (2019). The fatty acid oxidation enzyme long-chain acyl-CoA dehydrogenase can be a source of mitochondrial hydrogen peroxide. Redox Biol..

[23] Rajesh K (2016). The eIF2alpha serine 51 phosphorylation-ATF4 arm promotes HIPPO signaling and cell death under oxidative stress. Oncotarget.

[24] Ye J (2017). JCAD promotes progression of nonalcoholic steatohepatitis to liver cancer by inhibiting LATS2 kinase activity. Cancer Res..

[25] Brodowska K (2014). The clinically used photosensitizer Verteporfin (VP) inhibits YAP-TEAD and human retinoblastoma cell growth in vitro without light activation. Exp. Eye Res..

[26] Chen M (2017). Verteporfin inhibits cell proliferation and induces apoptosis in human leukemia nb4 cells without light activation. Int J. Med. Sci..

[27] Huggett MT (2014). Phase I/II study of verteporfin photodynamic therapy in locally advanced pancreatic cancer. Br. J. Cancer.

[28] Lu T (2018). The Hippo/YAP1 pathway interacts with FGFR1 signaling to maintain stemness in lung cancer. Cancer Lett..

[29] Broutier L (2017). Human primary liver cancer-derived organoid cultures for disease modeling and drug screening. Nat. Med..

