# Supplementary material for: ACADL plays a tumor-suppressor role by targeting Hippo/YAP signaling in hepatocellular carcinoma
Source: NPJ Precis Oncol. 2020 Mar 25;4:7. doi: 10.1038/s41698-020-0111-4 (PMC7096519; doi:10.1038/s41698-020-0111-4)
Supplement: Supplementary file 1 — Supplementary material [file 41698_2020_111_MOESM1_ESM.pdf]

Supplementary Table 1. Differentially expressed genes

Up-regulated (n=47)			Down-regulated (n=60)		
ACADL	TEAD1	TCEA1	AC007192.4	MAPK14	MMP14
MATR3	RAB7B	ZNF512	CLDN4	KCTD7	PITX2
ATRIP	YWHAB	CERS2	CTD-3222D19.2	SOX18	ELK3
CIPC	UGT1A9	TAOK3	ZNF260	ZNF267	PDK2
UGT1A3	PRDM2	CLSPN	CYR61	HOXB3	ZNF227
LHX2	ANKRD12	ZNF346	PRNP	MYD88	BLM
ZNF324B	ZNF17	NBN	ZNF284	SLC35F6	DUSP3
LIMS1	TADA2B		MDM4	ZNF224	H2AFJ
LIMS1	CDKN2C		ZKSCAN1	ZKSCAN8	CDIP1
RP11-2C24.9	UGT1A7		ZNF382	ZNF775	TGFBR1
SLC39A1	KLF7		IGFBP5	FRS2	TEAD3
SACS	CBX1		CANT1	RQCD1	CERS5
ZBTB3	BMP4		ZNF678	FOXM1	CCNA2
ZBTB43	CXCL8		CTGF	HIVEP1	ANKRD1
ZNF641	ZNF664		ADNP	TSN	USP47
TCF7	TCEAL1		ZNF155	FGFR3	SYF2
MAP1S	INSIG2		HSH2D	ZNF513	MRE11A
ZSCAN25	WFS1		STK4	PLA2G4A	CDK1
TOR1AIP1	IL18		ZNF527	NDRG4	RINT1
E2F3	CDKN2AIP		ALB	L3MBTL1	FANCI

Supplementary Table 2. Sequences of Real time-PCR primers

Gene	Forward Primer	Reverse Primer
ACADL	TTGGCAAAACAGTTGCTCAC	ACATGTATCCCCAACCTCCA
CCNA	CCAGAAACCCTTGCTGCATT	TGAGGGACACACACAGGTAC
CCNB1	TGGTGCACTTTCCTCCTTCT	TTAGCATGCTTCGATGTGGC
CCND1	GCATGTTCGTGGCCTCTAAG	CGTGTTTGCGGATGATCTGT
CCNE1	TCCTGGATGTTGACTGCCTT	TGTGTGCATCTTCATCAGCG
CDK1	TGGAGAAGGTACCTATGGAGTTG	AGCACATCCTGAAGACTGACT
CDK2	GGTGGTGGCGCTTAAGAAAA	ACCCGATGAGAATGGCAGAA
CDK4	AGTGTGAGAGTCCCCAATGG	CCTTGATCTCCCGGTCAGTT
CDK6	TGTTTCAGCTTCTCCGAGGT	TATGCAGCCAACACTCCAGA
CDKN1A	GGATGTCCGTCAGAACCCAT	GTGGGAAGGTAGAGCTTGGG
CDKN1B	GCAAGTACGAGTGGCAAGAG	CCAAATGCGTGTCCTCAGAG
CDKN2A	CTTCCTGGACACGCTGGT	TCAATCGGGGATGTCTGAG
CDKN2B	ACTAGTGGAGAAGGTGCGAC	ACCAGCGTGTCCAGGAAG
EPCAM	CAGAAGGAGATCACAACGCG	TCCAGATCCAGTTGTTCCCC
SOX2	AGCTCGCAGACCTACATGAA	CCGGGGAGATACATGCTGAT
KLF4	TCTCCCACATGAAGCGACTT	ATGGGTCAGCGAATTGGAGA
CTNNB1	CTTGTGCGTACTGTCCTTCG	AGTGGGATGGTGGGTGTAAG
NOTCH1	GGCCCTGAATTTCACTGTGG	GCCCTGGTAGCTCATCATCT
CTGF	TACCAATGACAACGCCTCCT	CCGTCGGTACATACTCCACA
CYR61	ATGGTCCCAGTGCTCAAAGA	GGGCCGGTATTTCTTCACAC
ANKRD1	TGAATCCACAGCCATCCACT	TCCTTCTCTGTCTTTGGCGT
18S	CGGCTACCACATCCAAGGAA	GCTGGAATTACCGCGGCT

Supplementary Figures and Legends

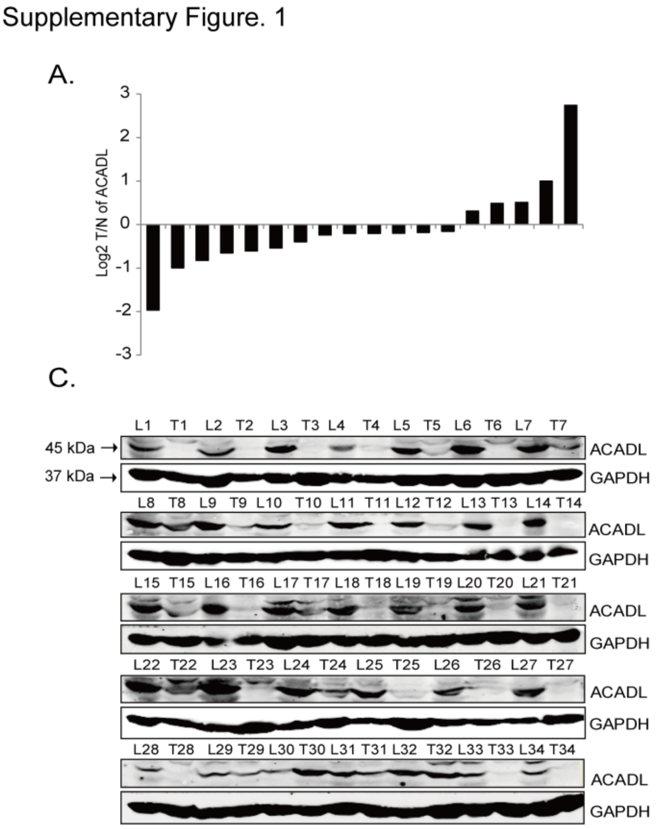

Supplementary Figure1. ACADL is downregulated in HCC.

(A&B) The expression levels of ACADL mRNA in 18 paired HCC (T) and adjacent non-tumor liver (L) tissues were determined by qRT-PCR (** $P < 0.01$).

(C&D) Expression of ACADL protein in 34 paired HCC and adjacent non-tumor liver tissues were examined by western blotting, normalized to GAPDH (*** $P < 0.001$).

Supplementary Figure. 2

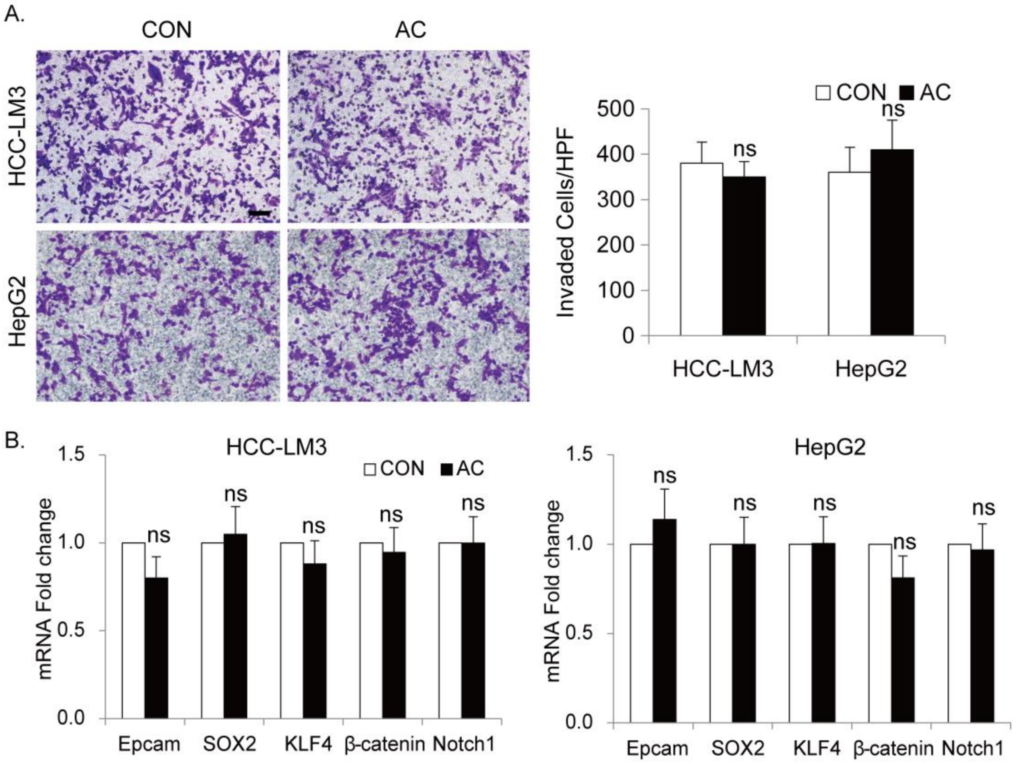

Supplementary Figure2. ACADL expression did not affect the cell migration and expression of stemness related genes.

(A) Indicated cells were seeded in Transwell and 24 hours later, the migrated cells were counted. Representative pictures were shown. Scale bar, 50 μm.

(B) The expression levels of EpCAM, SOX2, KLF4, β-catenin and NOTCH1 in control and ACADL overexpressing HCC-LM3 and HepG2 cell were analyzed by real time-PCR (ns=no significance).

Supplementary Figure. 3

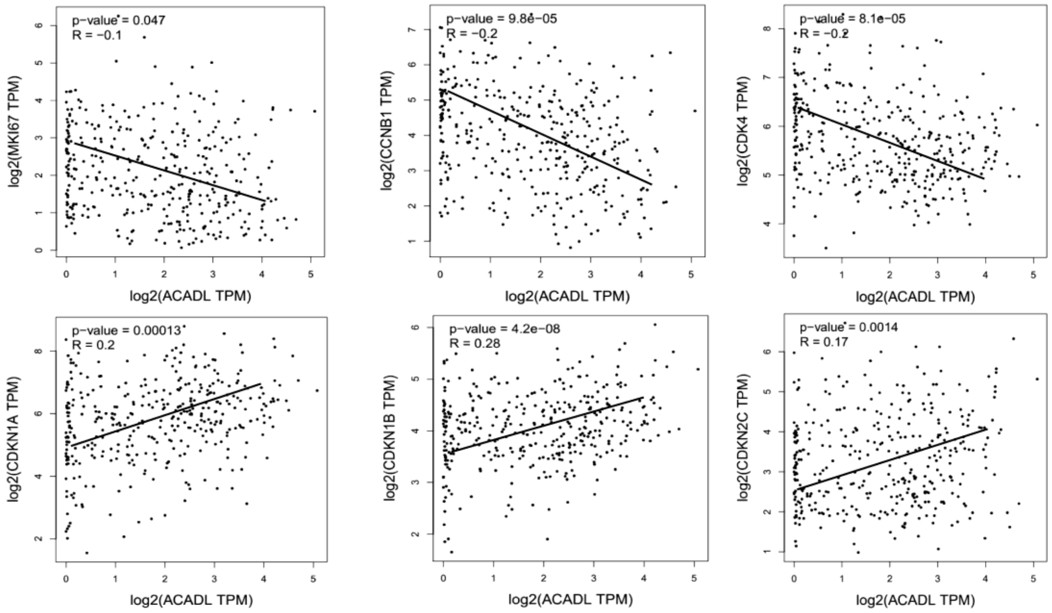

Supplementary Figure3. ACADL expression correlated with cell cycle regulators in HCC from TCGA.

Correlation analysis of ACADL and cell cycle related genes in HCC specimens from TCGA database.

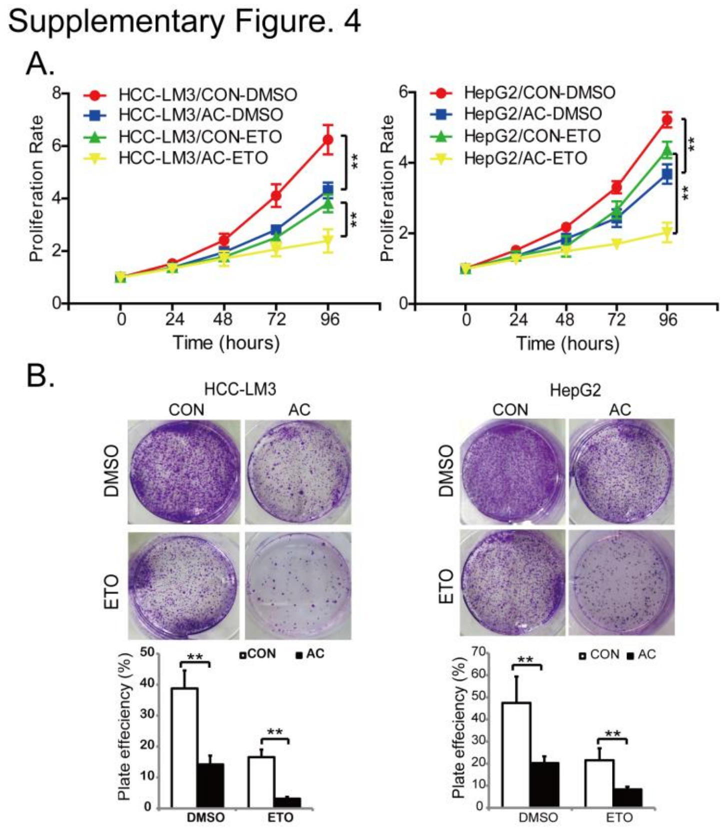

Supplementary Figure4. Blockage of fatty acid oxidation does not affect the growth inhibition effect of ACADL on HCC cells.

(A) The HCC-LM3/AC, HepG2/AC and control cells were treated with etomoxir (50 μM) or DMSO for indicated time. Cell proliferation was detected by CCK-8 assay at various time points (** P < 0.01).

(B) Representative images and quantification of colony formation of HCC-LM3 and HepG2 cells with indicated treatment (** P < 0.01).

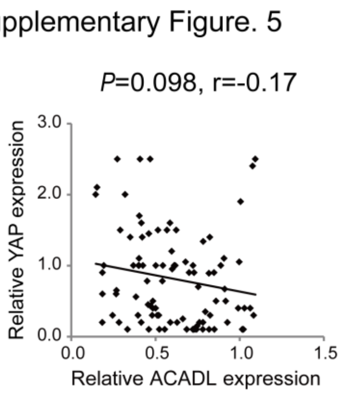

Supplementary Figure5. ACADL expression had no correlation with total YAP expression in HCC specimens.

The expression intensities of ACADL and YAP in 93 HCC specimens were quantified and

the correlation was calculated.
